# Resource utilization and costs of transitioning from pediatric to adult care for patients with chronic autoinflammatory and autoimmune disorders

**DOI:** 10.1186/s12969-024-00963-7

**Published:** 2024-02-23

**Authors:** Daniela Choukair, Christian Patry, Ronny Lehmann, Dorothea Treiber, Georg F. Hoffmann, Corinna Grasemann, Normi Bruck, Reinhard Berner, Peter Burgard, Hanns-Martin Lorenz, Burkhard Tönshoff

**Affiliations:** 1 Department of Pediatrics I, Center of Pediatrics and Adolescent Medicine, University Hospital Heidelberg, Heidelberg, Germany; 2 Center for Rare Diseases, University Hospital Heidelberg, Heidelberg, Germany; 3 Department of Pediatrics, St-Josef Hospital Bochum and Center for Rare Diseases, Ruhr-University, Bochum, Germany; 4 Department of Pediatrics, University Hospital Carl Gustav Carus, Technische Universität Dresden, Dresden, Germany; 5 Department of Oncology, Hematology and Rheumatology (Internal Medicine V), Heidelberg University Hospital, Heildelberg, Germany

**Keywords:** Chronic autoinflammatory and autoimmune disorders, Cost, Empowerment, Health literacy, Transition from pediatric to adult care

## Abstract

**Background:**

A structured transition of adolescents and young adults with chronic autoinflammatory and autoimmune disorders from the pediatric to the adult health care system is important. To date, data on the time, processes, outcome, resources required for the necessary components of the transition process and the associated costs are lacking.

**Methods:**

Evaluation of resource use and costs in a prospective cohort study of 58 adolescents with chronic autoinflammatory and autoimmune disorders, for the key elements of a structured transition pathway including (i) compilation of a summary of patient history, (ii) assessment of patients’ disease-related knowledge and needs, (iii) required education and counseling sessions, (iv) and a transfer appointment of the patient with the current pediatric and the future adult rheumatologist.

**Results:**

Forty-nine of 58 enrolled patients (84.5%) completed the transition pathway and were transferred to adult care. The mean time from the decision to start the transition process to the final transfer consultation was 315 ± 147 days. Transfer consultations were performed in 49 patients, including 10 patients jointly with the future adult rheumatologist. Most consultations were performed by the multidisciplinary team with a median of three team members and lasted 65.5 ± 21.3 min. The cumulative cost of all consultation and education sessions performed including the transfer appointment was 283 ± 164 Euro per patient. In addition, the cost of coordinating the transition process was 57.3 ± 15.4 Euro.

**Conclusions:**

A structured transition pathway for patients with chronic autoinflammatory and autoimmune disorders is resource and time consuming and should be adequately funded.

## Background

Chronic autoinflammatory and autoimmune disorders in childhood comprise a broad spectrum of diseases with different etiologies and clinical manifestations [1]. Despite the development of new treatment options, more than fifty percent of the patients require anti-inflammatory treatment into adulthood [2–5]. Therefore, a structured transition process is necessary for optimal treatment and prevention of long-term morbidity such as amyloidosis, osteoporosis and cardiovascular events [4, 6].

The key to successful long-term treatment is the transfer of the adolescents and young adults to the adult-centered health care system [7, 8]. The transition period for adolescents with chronic diseases occurs during a particular period of personal changes [9, 10]. Therefore, in about 40% of cases, the end of care in pediatric health care structures marks a break in medical care with negative consequences for adherence, deterioration of symptom control, or even the development of irreversible organ damage [11, 12]. To address these problems, several transition projects have been established worldwide, often designed for a specific disease or group of diseases and mostly at a local level [13]. For adolescents and young adults with juvenile-onset rheumatic and musculoskeletal diseases the Paediatric Rheumatology European Society (PReS) and the European League Against Rheumatism (EULAR) have developed a consensus statement focusing on patient education, empowerment, autonomy and multidisciplinary care [14]. Positive effects of a structured health care transition program with a multidisciplinary approach including psycho-social care and physiotherapy have been indicated by Boeker et al. [15], but these efforts are often underfunded in national health systems and therefore not widely or sustainably implemented [16, 17].

The German Federal Joint Committee (G-BA) funded the project TRANSLATE NAMSE from April 2017 to September 2020 to improve the care of patients with rare diseases (RD) [18, 19]. A consortium of ten German centers for RD, two health insurance companies (AOK Nordost; Barmer GEK) and the Alliance for Chronic Rare Diseases (ACHSE e.V.) was established to develop, test and evaluate a model for structured care of patients with RD [20]. The TRANSLATE-NAMSE project developed a generic pathway and additional tools that can be applied to adolescents with different RDs [21]. This pathway is based on previous experience with the more general “Berlin Transition Program” (BTP) [22]. This transition process was designed for a period of two years by providing a framework of transition consultations, and a structured summary of the disease course (summary of patient history). A case manager orchestrated the multidisciplinary care and ensured that the enrolled patients remained in the program, thus ensuring continuity of care. In the following we briefly describe the transition pathway and report the time required for the different components of the transition process and the corresponding personnel costs for the different members of the multidisciplinary team.

## Methods

A prospective cohort of 58 patients (16 males (28%)) with chronic autoinflammatory and autoimmune disorders was recruited between December 1, 2017 and February 28, 2020 at the Pediatric Rheumatology Outpatient Center of Pediatrics and Adolescent Medicine, University Hospital Heidelberg, Germany, of the University Hospital Carl Gustav Carus, Technische Universität Dresden, Germany, and of the University Hospital Essen, University Duisburg-Essen, Essen, Germany. This cohort was part of the nationwide health care project entitled TRANSLATE NAMSE funded by the Innovation Fund of the German Federal Joint Committee (G-BA), grant number 01NVF16024 TRANSLATE NAMSE [18, 19]. The Ethics Committee of the Charité, Berlin (#EA2/140/17) and the Ethics Committee of the University Hospital Heidelberg (S-499/2017) approved the study. Written informed consent was obtained from all parents/guardians, with assent from patients when appropriate for their age. Inclusion criteria were (i) chronic autoinflammatory and autoimmune disorders, (ii) age ≥ 16 years, (iii) willingness to participate in a structured transition program with expected transfer to adult care within 2 years. Exclusion criteria were (i) severe intellectual disability and (ii) lack of informed consent.

### Transition pathway

The transition pathway was developed as part of the national health care project TRANSLATE-NAMSE [21]. At the beginning of the transition process, patients were given a standardized questionnaire by their pediatric rheumatologist. This questionnaire was developed for this project, because validated questionnaires in German were not available. It contains 30 items on (A) disease-specific knowledge, (B) therapy-related knowledge, (C) social support and information, (D) future and career planning, (E) autonomous navigation through the medical system, and (F) wishes to the care team [23]. An Excel-based spreadsheet was used to quantify the current level of information and the need for education and counseling [23]. Based on this assessment, the case manager scheduled meetings with the multidisciplinary care team to provide health literacy education to patients in areas of identified knowledge and skill gaps. When indicated by the patient or deemed necessary by the pediatric rheumatologist additional appointments were scheduled for psychological, social-legal, and genetic counseling. 1–5 h of transition counseling were available per patient, depending on the need or request. Meanwhile, the attending pediatric rheumatologist provided a structured summary of patient history and recommendations for further management.

At the end of the transition process the case manager scheduled an appointment for the actual transfer of the patient to the adult health care system, preferably together with the pediatric and adult rheumatologists. This meeting included: (i) introducing the new care providers and structure, (ii) handing over all relevant patient information including molecular genetic results, (iii) explaining the adult care process, (iv) handing over the written summary of patient history to the adult rheumatologist, (v) providing the contact details of the adult care team in case of emergencies. If a transition consultation together with the adult care team was not possible, the future adult rheumatologist received the relevant documents and the important patient information was discussed with him/her by telephone. The transition process was completed with a final consultation in the pediatric clinic. A follow-up appointment was scheduled in the adult health care system to avoid losing contact with the patient. All items were documented in a checklist, including the initiation, duration, and completeness of this patient pathway, the quantified level of disease-specific knowledge, and the hours of education and counseling provided.

### Calculation of personnel costs

For the calculation of the personnel costs, the German personnel remuneration rates for the years 2017–2019 were used as presented in [24]. This resulted in the following costs per minute for the respective professional groups: pediatric or adult rheumatologist: 0.72 Euro/min; psychologist: 0.59 Euro/min; social worker: 0.52 Euro/min; nurse: 0.46 Euro/min. The cost of the transition consultation was calculated as follows: the cost in minutes of the participating members of the multidisciplinary team multiplied by the duration of the consultation in minutes. In addition, the cost of all consultations performed per patient was calculated as the sum of the costs of all transition consultations per patient. In addition, the average cost of a consultation per patient was calculated as the cost of all transition consultations per patient divided by the number of consultations.

### Statistical analysis

Patient data were collected in portable document format (PDF), which were read as comma-separated values (CSV) files and imported into SPSS 26 (SPSS Inv., Chicago, IL, USA), checked for plausibility and completeness, and analyzed descriptively. Data were tested for normal distribution using the Kolmogorov–Smirnov test. Data are presented as mean ± standard deviation (SD) or as median and range (minimum – maximum). Missing data were not included in the statistical analysis.

## Results

### Patient characteristics

**Fig. 1:**
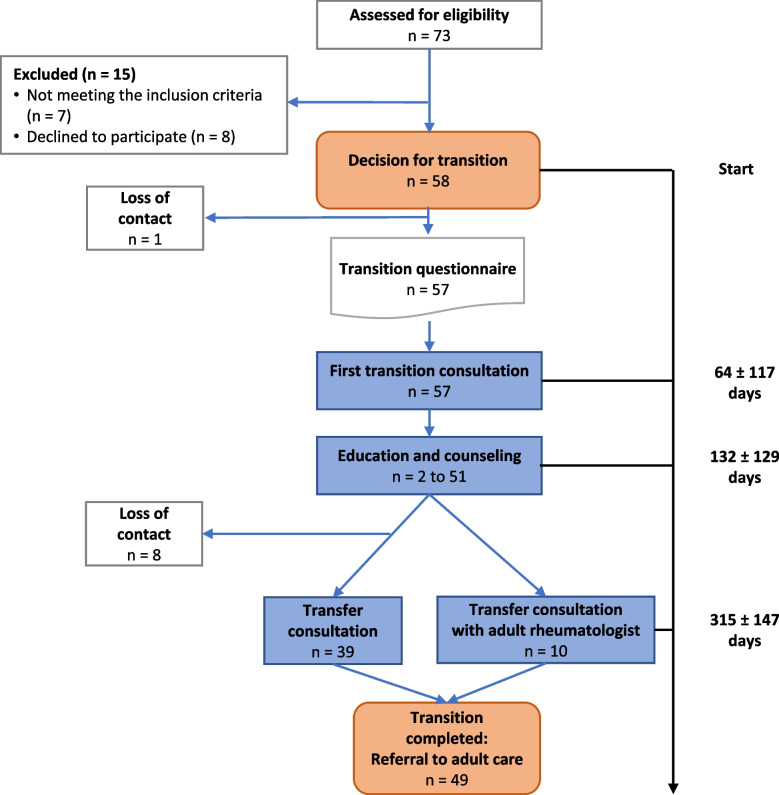
Flowchart of the transition pathway. See text for description. For education and counseling, *n* is the number of educational and counseling sessions provided. As shown in Figs. 3 and 4, the number ranged from 2 to 51

Patient characteristics and chronic autoinflammatory and autoimmune disorders are shown in Table 1. The mean age at enrollment was 17.5 ± 1.0 years. From a total of 73 patients, 58 patients (16 males, 42 females) with chronic autoinflammatory and autoimmune disorders were eligible according to the inclusion and exclusion criteria and underwent the structured transition pathway (Fig. 1). One patient had an autoimmune thrombocytopenia, one had chronic non-infectious osteomyelitis (CNO), and three had familial Mediterranean fever. Also included were 35 patients with juvenile idiopathic arthritis (JIA), two patients with chronic Lyme disease, six patients with non specified rare autoimmune diseases, nine with systemic lupus erythematosus, and one patient with non specified vasculitis (Table 1).

**Table 1:** Patient characteristics

Number of patients	58
Age at start of transition (mean years, SD)	17.5 (1.0)
Age at transfer to adult care (mean years, SD)	18.5 (1.0)
Male, female	16 (28%), 42 (72%)
Diseases	Frequency
Autoimmune thrombocytopenia	*n* = 1
Chronic non-infectious osteomyelitis (CNO)	*n* = 1
Familial Mediterranean fever	*n* = 3
Juvenile idiopathic arthritis (JIA)	*n* = 35
Chronic Lyme disease	*n* = 2
Non specified rare autoimmune disease	*n* = 6
Systemic lupus erythematosus	*n* = 9
Non specified vasculitis	*n* = 1

### Education and Counseling

#### Knowledge assessment

**Fig. 2:**
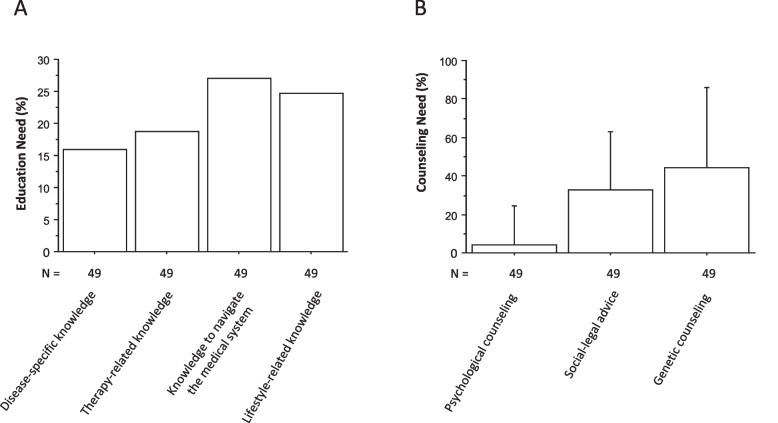
Educational (**A**) and counseling (**B**) needs of patients with chronic autoinflammatory and autoimmune disorders. N indicates the number of questions answered. Data are mean ± SD

Self-assessed knowledge gaps (as indicated by the responses ‘partially agree; disagree’) were reported by 16–27% of patients (Fig. 2 A). There were no significant differences between patients with different underlying autoinflammatory diseases (Table 1) for the items ‘disease-specific knowledge and need for education’, ‘therapy-related knowledge’, ‘ability to navigate the medical system independently` and ‘lifestyle-related knowledge’. The adolescents had a higher need for genetic counseling (44 ± 23%) than a self-assessed need for social-legal counseling (32 ± 20%) and a low self-assessed need for psychological counseling (4 ± 20%) (Fig. 2 B).

#### Educational sessions

**Fig. 3:**
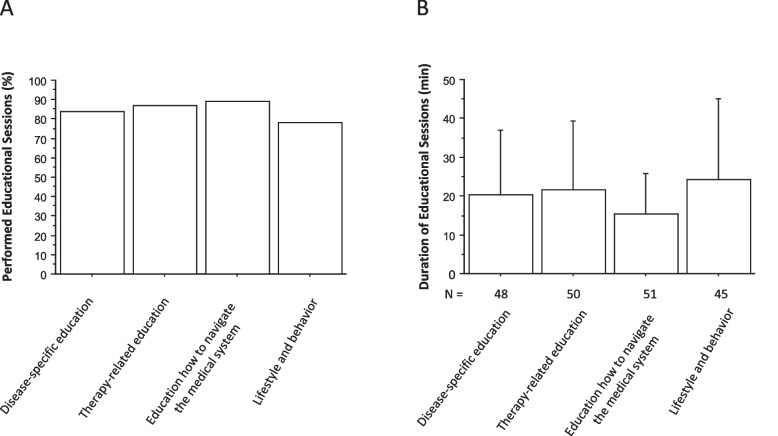
Number (**A**) and duration (**B**) of educational sessions (**B**) for patients with chronic autoinflammatory and autoimmune disorders. N indicates the number of educational sessions provided. Data are mean ± SD

A median of 3 (range 1–5) educational or counseling sessions were provided to patients with chronic autoinflammatory disease (Table 2). As assessed by the patient questionnaire, counseling was tailored to the individual needs of each patient. The number of counselors in the multidisciplinary team (physicians, psychologists, social workers, nurses) varied from 1 to 5 (median, 3 team members) (Table 2). Patients received education on disease and on disease-specific medications in 84% and in 87%, on navigating the medical system (independence) in 89%, and on lifestyle in 78% (Fig. 3 A). The average duration of each specific education session ranged from 15 to 24 min (Fig. 3 B).

**Table 2:** Resource consumption: number and duration of consultations, number of team members involved and time required for epicrisis preparation and coordination

	All transition consultations (*n* = 57)	Transfer consultations (*n* = 39)	Transfer consultations with adult rheumatologist (*n* = 10)
Number of consultations	Median 3 (range, 1–5)	1	1
Duration of consultations in minutes Mean (SD)	65.5 (21.3) (range, 15–150)	52.5 (24) (range, 10–100)	58.5 (14.9) (range, 30–90)
Number of team members involved	Median 3 (range, 1–5)	Median 3 (range, 1–5)	Median 3 (range, 2–4)
Administration	Time for preparation of summary of patient history (min)	Time for coordination (min)	
Mean (SD)	47.8 (17.0) (range, 30–120)	112.7 (25.8) (range, 50–240)

#### Counseling sessions

**Fig. 4:**
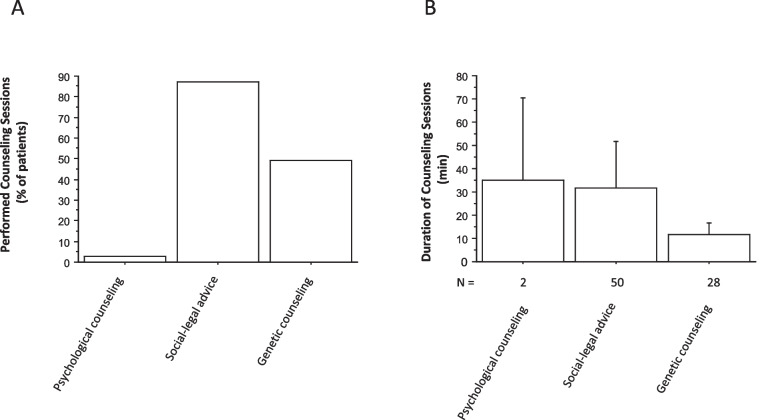
Number (**A**) and duration (**B**) of counseling sessions for patients with chronic autoinflammatory and autoimmune disorderss. N indicates the number of counseling sessions provided. Data are mean ± SD

The need to discuss legal aspects with adolescents suffering from chronic autoinflammatory disease was higher than the need to discuss genetic aspects (Fig. 4 A). The need for psychological counseling was quite low (3%) (Fig. 4 A). However, when performed in two patients, it took at least as long as social-legal counseling (mean, 35 ± 25 min; range, 10–60 min *vs.* mean, 31 ± 20 min; range, 5–120 min) (Fig. 4 B).

### Transfer to adult care

Of the 58 patients initially enrolled, 49 (84.5%) completed the entire transition pathway (Fig. 1). The mean age at transfer was 18.5 ± 1.0 years (range, 17–23.1 years) (Table 1). The mean time from the decision to start the transition process to the final transfer consultation was 315 ± 147 days (Fig. 1).

Transfer consultations were performed in 49 patients, in particular in 10 patients together with the future adult rheumatologist (Table 2). Most consultations were performed by the multidisciplinary team with a median of 3 team members. Consultations lasted 65.5 ± 21.3 min (Table 2). The mean duration of the transfer consultation with the future adult rheumatologist was 58.5 ± 14.9 min and without 52.5 ± 24 min (Table 2). The median number of participating multidisciplinary team members was three. At the end of the transition process, all patients were transferred to adult care (Fig. 1).

### Administration

A central document in the transition process is the preparation of a summary of patient history; the mean time required to prepare this summary of patient history was 47.8 ± 17 min (range, 30–120) (Table 2). The transition process is structured by the case manager. First, the distribution and later the evaluation of the questionnaires were essential to assess the educational needs. The case manager was also responsible for scheduling outpatient visits, inviting the necessary members of the multidisciplinary team, and communicating with the patients and their families. This organizational work took an average of 112.7 ± 25.8 min (range, 50–240) (Table 2).

### Costs of counseling sessions and team members

The mean cumulative cost of all counseling and education sessions necessary, including the transfer counseling, was 283 ± 164 Euro (Table 3). The cost per individual consultation was 89.4 ± 34.7 Euro. Regarding the costs of the multidisciplinary team members, the costs for the pediatric rheumatologist were the highest, followed by the costs for the psychologist, the nurse, the adult rheumatologist and for the social worker. The cost of writing the summary of patient history was 34.2 ± 12.3 Euro, and the cost of coordinating the transition process was 57.3 ± 15.4 Euro (Table 3).

**Table 3:** Cost of resources: costs of participating team members during consultations, cost of preparing the epicrisis and cost of coordination

	Total transition consultations	Per transition consultation	Transfer consultations	Transfer consultation with adult rheumatologist
Cost of consultations (Euro) Mean (SD)	283 (164) (range, 35–981) *n* = 57	89.4 (34.7) (range, 35.4–196) *n* = 57	75.8 (43.8) (range, 7.2–170)* n* = 39	109 (45.4) (range, 43.2–217)* n* = 10
Pediatric rheumotologist (Euro) Mean (SD)	140 (80.8) (range, 21–583)* n* = 57	44.5 (14.7) (range, 21–116)* n* = 57	37.4 (18.2) (range, 28.8–72)* n* = 39	45.1 (17.3) (range, 21.6–86.4)* n* = 10
Adult rheumotologist (Euro) Mean (SD)	67.6 (39.0) (range, 21–140)* n* = 10	18.9 (9.65) (range, 8.6–35.1)* n* = 10	0	41.2 (10.6) (range, 21.6–64.8)* n* = 10
Psychologist (Euro)	88.5*n* = 2	29.5*n* = 2	0	0
Nurse (Euro) Mean (SD)	82.5 (39.5) (range, 13–181)* n* = 53	26.2 (8.5) (range, 8.4–44.6)* n* = 53	25.5 (10) (range, 8.4–46)* n* = 34	29.6 (5.2) (range, 27.6–41.4)* n* = 7
Social worker (Euro) Mean (SD)	66.7 (44.9) (range, 15.6–218)* n* = 44	21.4 (12.6) (range, 4.6–52)* n* = 44	37.7 (9.9) (range, 26–52)* n* = 15	46.8 *n* = 1
Administration (Euro)	Cost of preparation of summary of patient history	Cost of coordination		
Mean (SD)	34.2 (12.3) (range, 21.6–86.4)* n* = 57	57.3 (15.4) (range, 26–125)* n* = 57		

## Discussion

To our knowledge, this is the first study to precisely quantify the time required for the different components of the transition process in patients with chronic autoinflammatory and autoimmune disorders. In addition, the total as well as the proportional personnel costs for the different members of the multidisciplinary team were estimated. In this cohort the transition process took between 100 and 776 days, with a mean of 315 ± 147 days. This is much shorter compared to the duration for kidney transplant recipients (624 ± 150 [range, 307–819] days) and for patients with pre-transplant CKD (365 ± 172 [range, 1–693] days) [24]. Administrative management of the transition took approximately two hours and was also less time consuming than for adolescents and young adults with chronic kidney disease [24]. This structured transition pathway for adolescents with chronic autoinflammatory and autoimmune disorders resulted in 84.6% of patients successfully transitioning to adult care, while 15.4% were lost to follow-up. At the end of this pathway, all patients received a transfer consultation performed by the multidisciplinary team with a median of 3 team members. In particular in 10 patients a transfer consultation was organized together with the future adult rheumatologist. In a survey by the European Reference Network on Immunodeficiency, Autoinflammatory, and Autoimmune Diseases Transition Working Group 68% of centers for systemic auto-inflammatory diseases had at least one joint appointment with pediatric and adult services prior to the transfer of care [25]. The relatively small number in our study could be explained by the fact that the majority of patients were transferred to adult rheumatologists in private practice who were not affiliated with a center.

At the beginning of the transition process a standardized questionnaire was used to assess counseling needs [21]. In this study, the self-assessed knowledge gaps were generally quite small, especially with regard to disease-specific and therapy-related knowledge. However, even when self-reported knowledge gaps are small, at least 78% of patients received education of all topics, with mean duration ranging from 15 to 24 min. Repeated and regular education is a crucial part of the transition process as it improves patient empowerment [26–29]; it is therefore recommended by the current consensus guidelines [14, 30, 31]. Empowerment is strongly associated with self-management, which improves health outcomes in chronic diseases not only by improving adherence to medications and recommendations, but also by increasing the individual’s ability to overcome challenges and manage problems independently [29]

In this cohort, only few requests for psychological counseling were reported in the questionnaire and it was provided to only two patients (3%). Compared to the whole transition cohort psychological counseling was offered in 16.4% of patients [23], and in kidney transplant recipients it was needed by 72% of patients [24]. Presumably, patients with autoinflammatory diseases are in a stable phase of their disease, feel quite well and mostly do not need psychological support.

A special interest of our study was to calculate the costs of the entire transition process, excluding standard care such as physical examination, laboratory tests, and apparative examinations that are part of these consultations. Consultations with other specialists, such as ophthalmologists or physiotherapists, were also not included in this calculation. The mean cumulative cost of all counseling and education sessions performed including the transfer counseling was 283 ± 164 Euro. This is less expensive than the whole transition process of the entire cohort of all chronically ill adolescents and young adults (599 ± 380 Euro) [32] or of kidney transplant recipients (966 ± 457 Euro) [24]. These differences can be explained by different patient population with different underlying diagnoses and therefore different complexity and severity of illness. Compared to the costs of other medical interventions, e.g. one day of inpatient care in a university hospital, this is a relatively small amount. Jensen et al. demonstrated that a social worker transition coordinator can significantly improve the rate of pediatric rheumatology patients who successfully transition to adult care [33]. In our study the transition process was structured by a case manager, and the cost per patient appear reasonable with 57.3 ± 15.4 Euros. The mean age at the start of the transition process was 18.5 ± 1.0 years, which may seem rather late. According to national and international consensus guidelines, transition should be initiated at 12—14 years of age in stable disease, and education and counseling as part of the transition process should begin at approximately 16 years of age [14, 25, 30]. Considering that the transition process takes 2—4 years longer, and that this would increase the costs by a factor of 2—4, the associated costs are still quite manageable. It is unreasonable that the costs of the transition are not yet covered by standard health insurance in many countries. Ultimately, a successful transition process is cost-effective, because increased disease activity with costly medical complications can be largely avoided. In Germany, there is still a lack of nationwide funding for the transition of adolescents with chronic diseases to adult care.

The strength of this study is that, for the first time, it evaluates a newly established transition pathway for patients with chronic autoinflammatory and autoimmune disorders and calculates the time and human and financial resources required for education, counseling, and transfer sessions. A limitation is the lack of long-term follow-up data, because the funding of the TRANSLATE-NAMSE project was limited to 3 years. Furthermore, this study was a study of three German centers; therefore, it is unlikely to be representative of all German centers or in a more global perspective. The calculated personnel costs are only valid for Germany and could be quite different for non-German countries. Outcome indicators of a successful transition should be evaluated as suggested by Fair et al. including individual, health care and social outcomes [34].

## Conclusions

This study shows that a structured transition pathway for patients with chronic autoinflammatory and autoimmune disorders is time and resource consuming due to the complexity of the diseases. Given that the costs are reasonable sustainable funding should be mandatory.

## Data Availability

Additional data are available upon request from the corresponding author if in line with the consents.

## Abbreviations

ACHSE: Alliance for Chronic Rare Diseases
BTP: Berlin Transition Program
CNO: Chronic non-infectious osteomyelitis
CSV: Comma-separated values
EULAR: European League Against Rheumatism
G-BA: Federal Joint Committee
PDF: Portable document format
PReS: Paediatric Rheumatology European Society
RD: Rare diseases
SD: Standard deviation

## References

[1] Krainer J, Siebenhandl S, Weinhäusel A (2020). Systemic autoinflammatory diseases. J Autoimmun.

[2] Minden K, Horneff G, Niewerth M, Seipelt E, Aringer M, Aries P (2019). Time of Disease-Modifying Antirheumatic Drug Start in Juvenile Idiopathic Arthritis and the Likelihood of a Drug-Free Remission in Young Adulthood. Arthritis Care Res (Hoboken).

[3] Minden K (2009). Adult outcomes of patients with juvenile idiopathic arthritis. Hormone Res.

[4] Blank N, Schönland SO (2020). Autoinflammatory syndromes and AA amyloidosis. Z Rheumatol.

[5] Ravelli A, Martini A (2007). Juvenile idiopathic arthritis. Lancet.

[6] Hersh A, von Scheven E, Yelin E (2011). Adult outcomes of childhood-onset rheumatic diseases. Nat Rev Rheumatol.

[7] Kashikar-Zuck S (2021). Transition of care for adolescents with chronic pain. Lancet Child Adolesc Health.

[8] Kordonouri O (2017). Transition of care for young adults with chronic diseases. Lancet Child Adolesc Health.

[9] Blum RW, Garell D, Hodgman CH, Jorissen TW, Okinow NA, Orr DP, et al. Transition from child-centered to adult health-care systems for adolescents with chronic conditions. A position paper of the Society for Adolescent Medicine. J Adolesc Health. 1993;14(7):570–6. doi: 10.1016/1054-139x(93)90143-d. 10.1016/1054-139x(93)90143-d 8312295

[10] Sawyer SM, Drew S, Yeo MS, Britto MT (2007). Adolescents with a chronic condition: challenges living, challenges treating. Lancet.

[11] Van Walleghem N, Macdonald CA, Dean HJ (2008). Evaluation of a systems navigator model for transition from pediatric to adult care for young adults with type 1 diabetes. Diabetes Care.

[12] Schütz L, Radke M, Menzel S, Däbritz J (2019). Long-term implications of structured transition of adolescents with inflammatory bowel disease into adult health care: a retrospective study. BMC Gastroenterol.

[13] While A, Forbes A, Ullman R, Lewis S, Mathes L, Griffiths P (2004). Good practices that address continuity during transition from child to adult care: synthesis of the evidence. Child Care Health Dev.

[14] Foster HE, Minden K, Clemente D, Leon L, McDonagh JE, Kamphuis S (2017). EULAR/PReS standards and recommendations for the transitional care of young people with juvenile-onset rheumatic diseases. Ann Rheum Dis.

[15] Boeker LS, Kuemmerle-Deschner JB, Saur SJ, Klotsche J, Erbis G, Hansmann S (2022). Health-related quality of life, continuity of care and patient satisfaction: long-term outcomes of former patients of the Tuebingen Transition Program (TTP) - a retrospective cohort study. Pediatr Rheumatol Online J.

[16] Bavisetty S, Grody WW, Yazdani S (2013). Emergence of pediatric rare diseases: Review of present policies and opportunities for improvement. Rare Dis.

[17] Pape L, Lämmermühle J, Oldhafer M, Blume C, Weiss R, Ahlenstiel T (2013). Different models of transition to adult care after pediatric kidney transplantation: a comparative study. Pediatr Transplant.

[18] Translate Namse. https://translate-namse.charite.de/en/ Accessed 20.07.2021.

[19] Gemeinsamer Bundesausschuss: TRANSLATE-NAMSE–Verbesserung der Versorgung von Menschen mit seltenen Erkrankungen durch Umsetzung von im nationalen Aktionsplan (NAMSE) konsentierten Maßnahmen. https://innovationsfonds.g-ba.de/projekte/neue-versorgungsformen/translate-namse-verbesserung-der-versorgung-von-menschen-mit-seltenen-erkrankungen-durch-umsetzung-von-im-nationalen-aktionsplan-namse-konsentierten-massnahmen.78 (2018). Accessed 20.07.2021.

[20] Grüters-Kieslich A, Burgard P, Berner R, Hoffmann G (2017). Zentren für seltene Erkrankungen. Monatsschrift Kinderheilkunde.

[21] Grasemann C, Matar N, Bauer J, Manka E, Mundlos C, Krude H (2022). Ein strukturierter Versorgungspfad von der Pädiatrie in die Erwachsenenmedizin für Jugendliche und junge Erwachsene mit einer seltenen Erkrankung. Monatsschrift Kinderheilkunde.

[22] Müther S, Oldhafer M, Siegmund B (2018). Transition medicine-structural solutions. Internist (Berl).

[23] Grasemann C, Höppner J, Burgard P, Schündeln MM, Matar N, Müller G (2023). Transition for adolescents with a rare disease: results of a nationwide German project. Orphanet J Rare Dis.

[24] Choukair D, Rieger S, Bethe D, Treiber D, Hoffmann GF, Grasemann C (2024). Resource use and costs of transitioning from pediatric to adult care for patients with chronic kidney disease. Pediatr Nephrol.

[25] Israni M, Nicholson B, Mahlaoui N, Obici L, Rossi-Semerano L, Lachmann H (2023). Current Transition Practice for Primary Immunodeficiencies and Autoinflammatory Diseases in Europe: a RITA-ERN Survey. J Clin Immunol.

[26] Acuña Mora M, Sparud-Lundin C, Bratt EL, Moons P (2017). Person-centred transition programme to empower adolescents with congenital heart disease in the transition to adulthood: a study protocol for a hybrid randomised controlled trial (STEPSTONES project). BMJ Open.

[27] Cooley WC, Sagerman PJ (2011). Supporting the health care transition from adolescence to adulthood in the medical home. Pediatrics.

[28] Anderson RM, Funnell MM (2010). Patient empowerment: myths and misconceptions. Patient Educ Couns.

[29] Bravo P, Edwards A, Barr PJ, Scholl I, Elwyn G, McAllister M (2015). Conceptualising patient empowerment: a mixed methods study. BMC Health Serv Res.

[30] Transitionsmedizin Gf: Transition von der Pädiatrie in die Erwachsenenmedizin. https://register.awmf.org/assets/guidelines/186-001l_S3_Transition_Paediatrie_Erwachsenenmedizin_2021-04.pdf Accessed 14–12–2022 2022.

[31] Sabbagh S, Ronis T, White PH (2018). Pediatric rheumatology: addressing the transition to adult-orientated health care. Open Access Rheumatol.

[32] Grasemann C, Höppner J, Burgard P, Matar N, Hoffmann GF, Müller G (2022). Ressourcenverbrauch der strukturierten Transition junger Menschen mit seltener Erkrankung aus der Pädiatrie in die Erwachsenenmedizin. Monatsschrift Kinderheilkunde.

[33] Jensen PT, Karnes J, Jones K, Lehman A, Rennebohm R, Higgins GC (2015). Quantitative evaluation of a pediatric rheumatology transition program. Pediatr Rheumatol Online J.

[34] Fair C, Cuttance J, Sharma N, Maslow G, Wiener L, Betz C (2016). International and Interdisciplinary Identification of Health Care Transition Outcomes. JAMA Pediatr.

